# Exploring Gender Differences in Gaming Among Italian University Students: A Discriminant Analysis

**DOI:** 10.3390/bs16071223

**Published:** 2026-07-18

**Authors:** Júlia Gisbert-Pérez, Claudio Longobardi, Manuel Martí-Vilar, Ali Ijaz, Laura Badenes-Ribera

**Affiliations:** 1 Faculty of Psychology and Speech Therapy, Universitat de València, Av Blasco Ibáñez, 21, 46010 Valencia, Spainmanuel.marti-vilar@uv.es (M.M.-V.); 2 Department of Psychology, University of Turin, Via Giuseppe Verdi, 10, 10124 Turin, Italyali.ijaz@unito.it (A.I.)

**Keywords:** gaming, gender, university, Italy, gaming motivations, perceived toxicity

## Abstract

Background: Gaming is a common leisure activity among university students, a life stage marked by late adolescence and emerging adulthood, when digital habits and psychosocial outcomes are still developing. However, gaming behaviors and motivations differ by gender, making it important to understand these variations to identify trends and potential risks linked to excessive use. This study examines whether gender is associated with differences in gaming habits, motivations, and perceived toxicity within a sample of Italian university gamers. Methods: A total of 394 participants (*M* = 22.8 years, *SD* = 3.6; 58% male) who actively played video games completed an online questionnaire on sociodemographic and gaming variables. Data were analyzed using *t*-tests and linear discriminant analysis. Results: Results showed gender differences in age of gaming onset, weekly gaming time, perceived toxicity, and motivations such as violent gratification, cognitive challenge, coping, and social interaction. However, discriminant analysis identified weekly gaming hours, violent gratification, and age as key differentiating factors, but the model accounted for a limited proportion of between-group variability. Conclusions: These findings suggest that gender accounts for a limited proportion of variability, indicating a trend toward gender parity in the analyzed gaming variables.

## 1. Introduction

Video games are immersive virtual environments in which players pursue goals and interact with complex systems (Jenkins, 2009). Despite the medium’s global growth, its history and culture have long been shaped by male dominance across the entire value chain of the industry. Women remain underrepresented in development teams and leadership positions, while men are disproportionately represented in highly visible competitive spaces such as esports (Kuss & Griffiths, 2012; Paaßen et al., 2017). As a result, the historical male orientation of gaming culture continues to shape contemporary perceptions of player behavior, including assumptions about gendered preferences, habits, and motivations (Lopez-Fernandez et al., 2019).

However, gaming culture is evolving due to increased accessibility and the diversification of gamer communities. The proliferation of platforms and expansion of game genres in recent years has made this digital leisure more inclusive. This has broadened the industry’s scope in terms of gender, geography and age. According to the Entertainment Software Association (2025), by 2025 women will represent 48% of the global gaming population. Furthermore, Italy is one of the most profitable gaming industries in Europe, beyond the largest global markets. According to Statista’s market research data (Statista, 2024), 63% of the Italian population actively engages in gaming. The most gaming-represented demographic in Italy is the 15 to 24 age group (IIDEA, 2024). In this context, late adolescence and emerging adulthood have become important stages in the development of the contemporary Italian gamer population.

Gender refers to self-defined gender identity regardless of individuals’ bodily attributes and sex assigned at birth (Lindqvist et al., 2021). Although gender encompasses a spectrum beyond the binary, research in this field has predominantly operationalised it in binary terms. In the context of gaming, gender identity may shape players’ behaviors, preferences, and self-identification as gamers through socialisation processes and group identification, as proposed by gender socialisation theory (Bem, 1993) and social identity theory (Tajfel & Turner, 1979). These theoretical frameworks suggest that gendered socialisation processes and identity formation may be associated with differences in gaming behaviors.

Despite increased female representation and progress in inclusivity, gender stereotypes and perceptions still influence gaming communities. On the one hand, many video games still feature female characters that are overly sexualised and objectified, which can create a hostile environment for women (López-Fernández et al., 2019). Conversely, women in certain gaming communities are exposed to sexist behaviors and gender-based violence (Belskie et al., 2023). Such experiences may affect their self-perception and influence their decisions about which communities to join or avoid. Consequently, these factors may affect how willing women are to identify as ‘gamers’. Despite the narrowing gap in participation rates, only 35% of women identify as gamers, compared to 51% of men (Entertainment Software Association, 2025; Le Ngoc, 2022).

Therefore, although the industry is moving towards gender parity in player statistics, certain aspects of video games and their communities continue to influence the gaming habits of men and women differently, as evidenced by studies in Spain (Gisbert-Pérez et al., 2024) and preliminary findings in Mexico (Gisbert-Pérez et al., in press). Variables studied in this field include gaming habits, player motivations, and the negative consequences of gaming.

Previous studies have identified distinct patterns in gaming preferences and levels of engagement by gender and sex. Studies consistently show that men are more engaged, resulting in a higher frequency of weekly gaming. For example, Miezah et al. (2020) found that males played for an average of 14 h per week, compared to 8 h for females. Similarly, Homer et al. (2012) observed even greater disparities, with males gaming for an average of 43 h per week (6 h per day), compared to 30 h per week (4 h per day) for females. Similarly, van Rooij et al. (2014) found that men spent an average of 15 h per week on online games, compared to 7 h for women. Furthermore, early exposure to video games has been linked to higher levels of engagement later in life (Nakayama et al., 2020), suggesting that the age at which people start gaming may influence their long-term gaming habits. However, research on gender differences in this area is limited.

Disparities in gaming frequency also appear to be reflected in the clinical prevalence of internet gaming disorder (IGD). Research indicates that risk patterns for IGD differ significantly by gender (Kuss et al., 2022). Stevens et al. (2021) conducted a meta-analysis of 53 studies published between 2009 and 2019 and identified a male-to-female prevalence ratio of 2.5:1. Notably, of the 31 studies that reported specific gender ratios, only three (9.6%) found a higher prevalence of IGD among female players. This suggests that higher engagement levels typically observed in males may be associated with increased vulnerability to problematic gaming behaviors.

Research indicates that gaming motivations and genre preferences are closely linked and vary by gender and region. According to a systematic review by Martucci et al. (2023), men typically play games for competitive reasons, whereas women often prioritise social and relational goals. However, these patterns showed geographic variability. In Europe, women often play to compete and demonstrate their skills (Lewis & Griffiths, 2011; Laconi et al., 2017), whereas in the United States, their motivations appear to be more closely linked to achievement, empowerment and social bonding. While women participate across a wide range of categories (Hassan et al., 2019), some studies suggest they prefer less aggressive or competitive genres than men do (Lange et al., 2021; López-Fernández et al., 2020).

This preference for specific genres may be a strategic response to the community environment, rather than indicating a lack of interest in competition. In certain competitive genres, the requirement for verbal communication can make women vulnerable to gender-based harassment, such as sexist comments, and toxic behavior (Belskie et al., 2023; Crothers et al., 2024). Toxicity refers to anti-social and/or disruptive behavior towards other gamers. These behaviors can range from offensive language during gameplay to trolling and hateful posts in game chats or on social media (Gandolfi & Ferdig, 2021). Such behaviors, particularly when gender-based, may cause women to avoid social online gaming, conceal their gender or abandon their ‘gamer’ identity. This limits their access to the social and psychological benefits of gaming (Kuss et al., 2022). Therefore, understanding the interplay between gaming motivations and external social barriers is crucial for grasping gendered gaming habits and the types of digital environments players choose to inhabit.

Similarly, several studies have examined women’s gaming behavior, producing mixed results (Kuss et al., 2022; Labrador et al., 2022; López-Fernández et al., 2019; McLean & Griffiths, 2019; Vermeulen et al., 2017). Given the inconsistencies observed in recent research, a more comprehensive investigation into gender differences among gamers is essential.

Gender differences identified in previous research may stem from methodological limitations, such as instruments lacking validation or reliability, or sociodemographic sampling variations, such as cross-national or broad age samples with high developmental variance (Hassan et al., 2019; Lange et al., 2021; Labrador et al., 2022). In addition, gaming practices are embedded in specific sociocultural contexts, and the gaming industry has developed unevenly across countries in terms of market expansion, platform accessibility, and the social visibility of gaming, which may shape both participation patterns and the meaning attached to gaming activities. As a result, patterns observed in one region may not fully generalise to others. To address these limitations, the present study adopts a twofold approach: (1) focusing on a specific demographic group within a single national context to minimise geographical and age-related variation, and (2) using a validated instrument with good internal consistency. Within this context, examining the extent to which gender relates to variability in gaming behaviors is relevant for understanding current gaming trends, deconstructing stereotypes and fostering more inclusive gaming communities. Beyond its social implications, this perspective may also inform game design, enabling experiences that cater to the diverse needs of all players.

### Aim of the Study

This study aims to examine whether gender is associated with differences in gaming habits, motivations, and perceived toxicity within a sample of Italian university gamers. Specifically, it examines gaming habits, motivations, and community-related variables in a sample of Italian university students. Italy represents a relevant context for this question because gaming is widespread among Italian young adults (De Pasquale et al., 2020). Due to the geographical variation in gaming participation (Visual Capitalist, 2025), it is important to establish whether global trends remain consistent within specific sociodemographic contexts. Therefore, this research analyzes whether the gaming evidence reported in previous research also applies to an Italian university sample. By focusing on this specific demographic, the study seeks to contribute additional evidence regarding gender-related differences in gaming experiences among university students.

The university years represent a critical developmental transition from late adolescence to emerging adulthood (Arnett et al., 2014). This period is characterized by increasing independence and engagement with digital spaces. However, it is also a period of instability and vulnerability, which can increase the likelihood of developing Internet Gaming Disorder (IGD; Kinghorn et al., 2018). In line with this, research conducted in Italy suggests that a significant proportion of university students may be at risk of IGD, with around 15% potentially meeting the full diagnostic criteria (De Pasquale et al., 2018). Therefore, examining the gaming habits, motivations and perceived toxicity of gaming within this population could contribute to the existing literature on gender-related differences in gaming engagement and experiences during this developmental stage.

Based on the aforementioned theories and prior research, the following theory-informed directional hypotheses were proposed:

**H1.**  *Men will report an earlier age of gaming onset.*

**H2.**  *Men will report more hours spent gaming per week.*

**H3.**  *Men will report gaming in communities with higher levels of perceived toxicity.*

**H4.**  *There will be statistically significant differences in gaming motivations between genders.*

**H5.**  *Gaming habits and motivations are discriminant factors between genders.*

## 2. Materials and Methods

### 2.1. Procedure and Sample

A cross-sectional study was carried out using a LimeSurvey (version 6.16.10) online survey administered in university classrooms. Participants were recruited between 2022 and 2024 through non-probabilistic convenience sampling, selected based on their accessibility and availability within the university environment. The recruitment process began with in-person invitations during face-to-face classes attended by students enrolled in different undergraduate programs. Participants were recruited as part of a broader research project on leisure and consumption habits among university students. Participation in the study was voluntary, and all participants provided informed consent before data collection. No incentives were offered to minimize potential self-selection bias. Additionally, participants were assured that their responses would remain anonymous to reduce the likelihood of social desirability bias. This study adhered to the ethical principles outlined in the 1964 Declaration of Helsinki and its subsequent amendments or comparable ethical standards. Ethical approval was obtained by the Institutional Review Board of the University of Turin (Protocol No. 0592473).

For the purposes of the present study, analyses were restricted to Italian-speaking university students aged 18 years or older who reported currently playing video games. The original sample consisted of 402 participants, with a response rate of 98%. Gender was treated as a binary variable, and only participants identifying as men or women were retained in the final sample. The final sample included 394 university gamers (*M*_age_ = 22.81, *SD*_age_ = 3.67, range = 18–34). The 42% of the sample identified as women.

### 2.2. Measures

Sociodemographic information. Participants provided sociodemographic information such as age, gender, and family income.

Gaming information. Participants provided gaming information such as age of gaming onset, weekly hours of gaming, and the level of perceived toxicity within their gaming communities (*how toxic do you consider the community or players to be in the games you usually play?*), ranging from 1 (absence of toxicity) to 5 (highly toxic).

Motivations for gaming. The e-MUV scale (López-Fernández et al., 2019) was administered to measure gaming motivations. This scale comprises 32 items assessing eight gaming motivations: immersion (items 1–4), customization (items 5–8), violent gratification (items 9–12), coping (items 13–16), fun (items 17–20), cognitive challenge (items 21–24), competition (items 25–28), and social interaction (items 29–32). Items were rated on a five-point Likert scale, from 1 (*strongly disagree*) to 5 (*strongly agree*). Higher scores indicated higher motivation for gaming in each specific gaming motivation. The Italian version of the e-MUV scale was based on a previous standardized back-translation adaptation and pilot testing (Gisbert-Pérez et al., 2024; Wild et al., 2005). Prior to the main analyses, a confirmatory factor analysis (CFA) was conducted to test the hypothesized 8-factor structure of the scale, showing an adequate model fit (χ^2^(436) = 1395.29, *p* < 0.001, CFI = 0.95, TLI = 0.94, RMSEA = 0.07, SRMR = 0.06). The Cronbach’s alpha (α) and McDonald omega reliability coefficients (ω) for the e-MUV dimensions ranged from 0.83 to 0.91.

### 2.3. Statistical Analysis

Descriptive statistics were initially computed to summarize the demographic characteristics and study variables. For continuous variables, means, standard deviations, and range were calculated, while categorical variables were summarized using frequencies and percentages. Additionally, skewness and kurtosis were examined to assess the normality of continuous variables (Kline, 2022).

Group comparisons assume the instrument’s psychometric properties are equivalent across gender groups. To ensure this equivalence, we conducted a differential item functioning (DIF) analysis using ordinal logistic regression (OLR; Zumbo, 1999). This method is suitable for small samples and does not require parametric assumptions of the shape of the construct distribution or item response (Belzak, 2020). To accurately capture the latent trait of each item in the e-MUV’s multidimensional structure, subscale-specific rest scores were utilized for matching. Research indicates that relying on a total test score in multidimensional assessments may inflate false-positive DIF detection, potentially misrepresenting the constructs measured (Mazor et al., 1998). OLR uses the likelihood ratio χ^2^ test and an effect size estimator to model three models: M1 (effect of dimension total score, T), M2 (T + gender), and M3 (T × gender). The DIF detection tests were Δ χ^2^, for global DIF detection (M3-M1 χ^2^), non-uniform DIF (M3-M2 χ^2^) and uniform DIF (M2-M1 χ^2^). The statistical significance criterion was *p* < 0.0003 (Bonferroni correction: 0.01/32 items; J. Kim & Oshima, 2013). Effect size criterion was the difference between Nagelkerke-*R*^2^ of each model compared (Δ*R*^2^) in three classifications (Jodoin & Gierl, 2001): negligible (<0.035), moderate (≥0.035), and large (≥0.070). The uniform (M2-M1) and non-uniform (M3-M2) DIF tests were conditional on the statistical significance and effect size of the overall DIF test (M3-M1).

After verifying that all relevant statistical assumptions were satisfied, gender differences across the study variables were then examined using independent samples *t*-tests. Hedges’ g was calculated to estimate effect sizes, providing an unbiased measure of the standardized mean difference. Following Lovakov and Agadullina (2021), effect sizes of 0.15, 0.36, and 0.65 were interpreted as small, moderate, and large, respectively. Given the number of motivation comparisons, results were additionally verified against Bonferroni- and FDR-corrected thresholds (see Table 1 note).

A linear discriminant analysis (LDA) was conducted using the forward stepwise selection method (Tabachnick & Fidell, 2014) to identify the gaming-related variables that best distinguished between men and women (H5). Only those variables that had shown a statistically significant association with gender in previous analyses were included as predictors. This selection method was used to derive a parsimonious exploratory model, which was particularly useful given the large number of variables under consideration. Unlike univariate tests, LDA identifies the linear combination of predictors that best separates the two gender groups and quantifies its overall discriminatory power, providing a more comprehensive multivariate perspective than isolated group comparisons. However, it is important to note that stepwise procedures can yield solutions that are sensitive to the sample, potentially leading to sample-dependent outcomes. The stepwise procedure added predictor variables sequentially based on the criterion of minimizing Wilks’ Lambda (tolerance < 0.001; entry *F* = 3.84; removal *F* = 2.71), thereby optimizing the discriminant function by reducing within-group variance and maximizing between-group variance. The resulting discriminant function was then employed to classify participants into their respective gender groups. Collinearity diagnostics revealed that all VIF values were below 10 and tolerance values were above 0.1, indicating no significant multicollinearity among the predictors.

Different procedures were used to evaluate the fit and performance of the discriminant function (Tabachnick & Fidell, 2014). First, Wilks’ Lambda and the χ^2^ significance test were applied to determine whether the group centroids differed significantly. Second, Canonical *R*^2^ was calculated to quantify the proportion of variance in group differences explained by the discriminant function. Third, the predictive accuracy of the function was assessed by examining the percentage of correctly classified cases. Finally, the stability, quality, and generalizability of the discriminant function were evaluated using a “leave-one-out” cross-validation procedure. A priori power analysis using G*Power 3.1 indicated that a minimum sample size of 374 participants was required for the LDA, assuming a moderate effect size (f^2^(V) = 0.0625; α = 0.05, power = 0.95). Descriptive statistical analyses, independent-samples *t*-tests, and the linear discriminant analysis were conducted with SPSS 28 (IBM Corp., 2021). R packages *lavaan* (Rosseel, 2012), *MASS* (Venables & Ripley, 2002), and *dplyr* (Wickham et al., 2023) were used for confirmatory factor analysis, differential item functioning analyses, and data management, respectively.

## 3. Results

### 3.1. Descriptive Statistics

The sample comprised 228 men (57.9%) and 166 women (42.1%). Participants’ mean age of gaming onset was 8.31 years old (*SD* = 3.12, range 3–23), with a mean weekly gaming time of 10.8 h (*SD* = 9.7, range 1–50). Regarding monthly family income, 4.1% (*n* = 17) reported less than 1000 €/month, 30% (*n* = 118) reported between 1001 and 2000 €/month, 26.2% (*n* = 103) reported between 2001 and 3000 €/month, 24.4% (*n* = 96) reported between 3001 and 4000 €/month, and 15.2% (*n =* 60) reported more than 4000 €/month.

In terms of toxicity levels, the data indicates that 19.8% (*n* = 78) of participants perceived low or no toxicity, 21.1% (*n* = 83) perceived low to medium levels of toxicity, 25% (*n* = 99) perceived medium levels, 17.8% (*n* = 70) reported high-medium levels, and 6.3% (*n* = 25) identified high levels of toxicity. Additionally, 9.9% (*n* = 39) of participants reported no toxicity in their gaming communities.

### 3.2. Differential Item Functioning

DIF analysis using subscale-specific rest scores as matching variables showed that only one item (item 1, Immersion) remained statistically significant after Bonferroni correction (Δ*R*^2^ = 0.0405), indicating moderate DIF. All remaining items show no evidence of meaningful uniform or non-uniform DIF. Therefore, a sensitivity analysis was conducted to check whether the results would be affected by removing item 1. No gender differences were found, effect sizes were negligible, and both versions were highly correlated (*r* = 0.98). These results indicate that the DIF item does not meaningfully affect the results.

### 3.3. Gender Differences in Study Variables

Table 1 includes Student’s *t*-test results examining gender differences in age and gaming variables included in the study. A statistically significant difference was found in the age distribution by gender, with women being younger in this sample (*M*_men_ = 23.36; *SD*_men_ = 3.67; *M*_women_ = 22.07; *SD*_women_ = 3.56; *p* < 0.001).

Regarding gaming variables, statistically significant differences by gender were observed in age of gaming onset (*p* = 0.008), weekly gaming hours (*p* < 0.001), and perceived toxicity of the community gaming (*p* < 0.001). Men began gaming at earlier ages than women (*M*_men_ = 7.96, *SD*_men_ = 2.96; *M*_women_ = 8.8, *SD*_women_ = 3.26), reported spending more hours gaming per week (*M*_men_ = 12.36, *SD*_men_ = 10.47; *M*_women_ = 8.66, *SD*_women_ = 8.18), and reported higher levels of perceived toxicity in the gaming communities in which they participated than women, as measured by a single self-report item (*M*_men_ = 2.59, *SD*_men_ = 1.39; M_women_ = 2.14, *SD*_women_ = 1.38).

**Figure 1:**
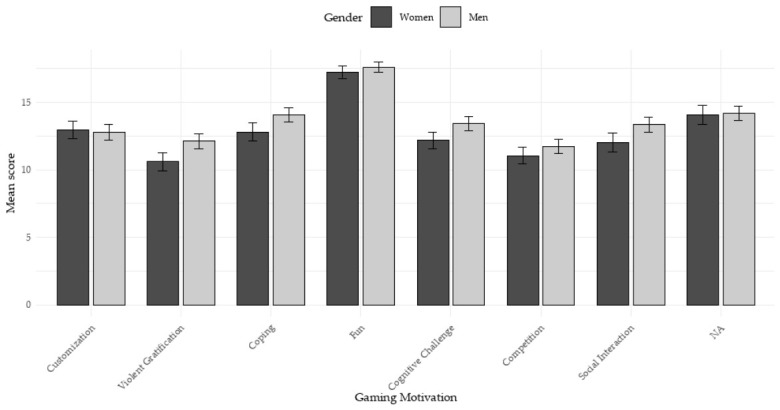
Mean distribution of gaming motivations by gender.

Regarding gaming motivations, statistically significant differences were found in violent gratification (*p* < 0.001), coping (*p* = 0.004), cognitive challenge (*p* = 0.003), and social interaction (*p* = 0.002; Figure 1). Men showed higher levels of all of these motivations compared to women. No statistically significant differences were found in the other gaming motivations.

### 3.4. Linear Discriminant Analysis

A single discriminant function was derived as only two gender groups were analyzed. Prior to the analysis, the assumption of equality of covariance matrices was checked with Box’s *M* test (Box *M* = 20.28, *F =* 3.35, *p* = 0.003), indicating a violation of the assumption. Box’s *M* test is highly sensitive to large sample sizes and frequently yields significant results even when the departures are minor. Thus, this problem was not considered problematic due to the large and relatively balanced group sizes (Tabachnick & Fidell, 2014), as the linear discriminant analysis remains sufficiently robust against this violation without distorting classification rates.

The discriminant function demonstrated a statistically significant association between the groups and all predictor variables, accounting for 8.7% of the between-group variability. The overall Wilks’ lambda was statistically significant (Wilks’ lambda = 0.91, *χ*^2^(3) = 35.44, *p* < 0.001), indicating that the discriminant function showed statistically significant but modest differentiation between the gender groups. Men had higher discriminant function scores (*M* = 0.26) compared to women (*M* = −0.36), and this difference was statistically significant (*t*(392) = −6.06, *p* < 0.001, Hedges’ *g* = 0.62, 95% CI: 0.41, 0.82).

Regarding the predictor variables, the correlation coefficients represent Pearson correlations between the predictors and the discriminant function (Table 2). Notably, all coefficients for the variables included in the model exceeded 0.50, surpassing the commonly accepted threshold of 0.30 for relevance in discriminant analysis. The strongest relationships with the discriminant function were observed for hours of weekly gameplay (*r* = 0.616) and violent gratification (*r* = 0.577), followed by age (*r* = 0.563). Specifically, higher levels of weekly gameplay, greater motivation for gratification, and being older were associated with higher discriminant function scores, which characterize the male group in this sample.

The overall accuracy rate was 62.7%, correctly classifying 62.7% of respondents into “male” or “female” groups. Women were classified with higher accuracy (66.3%) than males (60.1%; Table 3). The consistency between the original classification (62.7%) and cross-validation (61.7%) indicates similar, although modest, classification performance under cross-validation.

## 4. Discussion

This study aimed to explore gender differences in gaming characteristics. Overall, the findings indicate that, although several differences between men and women were observed, gaming habits, motivations and experiences were largely shared across genders. While previous research has documented gender-related differences in gaming alongside the evolving demographic profile of gamers and the persistence of exclusionary behaviors in some gaming communities (Kuss et al., 2022), the differences observed in this Italian university sample were generally small to moderate in magnitude.

Regarding H1, Italian male gamers reported starting gaming earlier than their female counterparts. These findings align with previous studies, which attribute earlier gaming onset in men to leisure activities historically designed for and promoted among males (López-Fernández et al., 2019). In line with H2, men also reported playing nearly four hours more on average. Findings from Nakayama et al. (2020) suggest that an earlier gaming onset is often associated with higher engagement in gaming, supporting H1 and H2.

Regarding H3, men reported somewhat higher perceived toxicity in their gaming communities than women, although this finding relies on a single self-report item of unknown reliability and should therefore be interpreted with caution. This result is consistent with the possibility that men more frequently participate in games with strong competitive and/or violent components (Lange et al., 2021; Bopp et al., 2022). Additionally, competitive or violent gaming communities may be associated with a higher risk of gender-based harassment. Consequently, some female gamers may choose alternative gaming contexts where there is less risk of exposure to harassment or violence, regardless of their gaming interests (Belskie et al., 2023; Kuss et al., 2022). However, because our toxicity item captures general perceived community toxicity rather than personally experienced harassment, this explanation remains speculative rather than directly tested by our data. Nevertheless, the observed effect size indicates a small difference, suggesting caution in interpreting this finding as a strong gender-based difference.

Regarding H4, statistically significant differences were found only in a limited number of gaming motivations between men and women. Specifically, men reported significantly higher scores in violent gratification, coping, and social interaction. These results are partially consistent with those found among Spanish university students, where men also showed higher scores in violent gratification, social interaction, and cognitive challenge (Gisbert-Pérez et al., 2024). Previous studies suggest that male gamers’ motivations are often associated with specific game genres, such as shooters or massively multiplayer online role-playing games (N. R. Kim et al., 2016). Similarly, women may prefer contexts where aggressive components or social interactions do not make them feel exposed (Belskie et al., 2023).

Moreover, motivation for cognitive challenge was also statistically higher in men than in women in this sample. Some gaming environments often reinforce competence- and strategy-based identities among male players, as stereotypical “gamer” traits are strongly associated with skill and performance, which can strengthen men’s identification with the gamer role (Yao et al., 2022). In this context, cognitive challenge may also function as a way of demonstrating gaming ability and status within the gaming environment. However, no differences were observed in competition-oriented motivations, which focus on competing against others. Since previous studies with Spanish university students and young gamers did find differences in both motivations (Demetrovics et al., 2011; Gisbert-Pérez et al., 2024), the discrepancy with the present findings may reflect characteristics specific to the sample or sociocultural context examined. Although all four differences remained statistically significant after Bonferroni and FDR correction, their small-to-moderate effect sizes warrant a cautious interpretation. These results may reflect a stronger tendency toward self-competition in the present sample, although this interpretation remains tentative and may also be influenced by broader sociocultural factors.

Finally, no statistically significant differences were found in other gaming motivations, such as immersion, customization, and fun, suggesting that these may represent common motivational components among Italian university students regardless of gender. Although customization has traditionally been associated more with female gamers (López-Fernández et al., 2020), these three motivations have shown lower risk in relation to gaming-related problems (Slack et al., 2022; Wang & Cheng, 2022).

The findings related to H1–H4 are consistent with expectations derived from gender socialization theory (Bem, 1993). This theory suggests that from an early age, boys and girls socialize differently regarding their interests and activities. Due to cultural and social stereotypes, video games are often more closely associated with male interests. Similarly, social identity theory (Tajfel & Turner, 1979) posits that individuals tend to identify with groups that reinforce their sense of self. Men may be particularly attracted to certain video games because they provide opportunities to express and consolidate masculine identity, particularly in genres emphasizing competition and action. This tendency to seek out competitive and action-oriented games may partly account for the higher presence of men in gaming communities perceived as more toxic, where aggressive behaviors are more common. At the same time, the relatively small effect sizes observed across the analyses suggest that these theoretical frameworks may help explain specific patterns of behavior, while substantial similarities remain between men and women in their gaming experiences.

Regarding H5, the discriminant analysis showed that weekly gaming hours, violent gratification, and participants’ age significantly differentiated between genders. Several cautious interpretations can be drawn from these findings. Weekly gaming hours and violent gratification may reflect behavioral patterns that were somewhat more common among men in the present sample, although the relatively small explained variance suggests substantial overlap between genders. Spending more time playing may indicate greater practice and a stronger drive to master gaming skills, while higher violent gratification scores may reflect the influence of gendered socialization processes. Age, on the other hand, captures demographic differences between the groups and serves as an additional contextual factor rather than a direct indicator of gaming behavior. Additionally, the relatively low proportion of explained variance by the discriminant function suggests that many gaming habits and motivations are shared across genders and that there is considerable within-group variability, potentially influenced by psychosocial factors such as individual needs (Przybylski et al., 2010) or social and contextual influences (Angelini et al., 2023). While research with Spanish university students found that gaming habits and motivations accounted for 36% of variance (Gisbert-Pérez et al., 2024), the present findings suggest that, in this Italian sample gender accounts for a more limited proportion of variability. This indicates that gender differences in gaming generally modest and that most gaming habits and motivations are shared across men and women university players.

### 4.1. Limitations and Future Research

This study has several limitations. First, sampling bias may have affected the study, as participation depended on voluntary access to the questionnaire and coverage by academic faculties was not recorded. This may have resulted in an uneven representation of faculties, favouring individuals with easier access to digital devices or a greater interest in gaming-related topics. Thus, the sample had a higher proportion of males and may not fully reflect the broader Italian university population, potentially affecting its representativeness. Second, the sample consisted of Italian university students, limiting the generalizability of the findings to other age groups or populations outside of an academic context. Third, gender was treated as a binary variable which limits the generalizability of the findings to gender-diverse individuals. Fourth, the cross-sectional design prevents drawing causal conclusions about the relationships between gender, gaming habits, motivations, and perceived toxicity. Fifth, the model did not include other relevant variables that may influence gaming behavior or may act as confounders, such as personality traits, specific game genres, or other psychosocial factors. Sixth, gaming information variables and toxicity were measured using a single-item measure. Although internal consistency is not applicable to these individual indicators, their test–retest reliability and criterion or construct validity were not examined. This limitation is especially relevant for perceived toxicity, a multifaceted construct that may not be adequately captured by a single item. Therefore, findings relating to toxicity should be considered exploratory. Seventh, measurement invariance of the eMUV scale across gender groups was not tested, which limits the ability to ensure that the construct is interpreted equivalently across groups. Finally, the study relied on self-report measures, which may be influenced by social desirability and introduce measurement error.

Future research should include more diverse samples, including gamers from different age groups, to examine whether gender-related predictors of gaming behavior remain stable across the lifespan. Cross-national studies will help to confirm whether findings are similar or different across countries. Future studies should examine the mechanisms underlying gender differences in gaming and the potential moderating effects in specific contexts. Ensuring measurement invariance of the eMUV scale across gender groups will also ensure equivalent measurement across genders. Future research could also benefit from incorporating alternative theoretical frameworks, such as Self-Determination Theory, to further explore intrinsic and extrinsic motivational processes underlying gaming behavior. Finally, further work is needed to develop and validate measurement instruments specifically tailored to gaming-related constructs, such as perceived toxicity within gaming communities.

### 4.2. Practical Implications

Some practical implications can be drawn, particularly for Italian university students, although they should be interpreted in light of the modest effect sizes observed. First, the presence of shared gaming motivations across genders suggests that game developers may benefit from designing experiences that appeal to a broad range of players, emphasizing elements such as immersion, enjoyment, and customization. Second, although men perceived somewhat higher toxicity in their gaming communities—a finding based on a single-item measure and therefore interpreted with caution—the higher perception of toxicity in certain gaming environments highlights the importance of fostering inclusive and respectful online communities across user groups. Within university settings, educational initiatives that promote awareness of respectful interaction and gender inclusion could further support healthier community dynamics. Finally, although differences in gaming intensity and motivation for violent gratification were observed at the group level, these patterns should be interpreted as preliminary indications that can inform more nuanced and context-sensitive approaches to prevention, where gender is considered alongside other individual and contextual factors.

## 5. Conclusions

This study examined gender differences in gaming habits, motivations and perceived toxicity among Italian university students. Overall, the findings suggest that, although some gender differences exist, particularly in gaming intensity and motivations such as violent gratification, there is substantial overlap in most gaming behaviors and motivations across genders. These results suggest that gender plays a limited role in explaining gaming patterns among university students, with most behaviors and motivations being widely shared. From a practical perspective, this suggests that efforts to promote inclusive gaming environments should focus less on gender segmentation and more on addressing shared user needs and reducing toxic behaviors that affect the gaming community.

## Figures and Tables

**Table 1:** Sociodemographic and gaming variables by gender.

	Men		Women						
	*M*	*SD*	*M*	*SD*	*t*	*df*	*p*	*g*	95% CI
Age	23.36	3.67	22.07	3.56	−3.47	392	<0.001	0.35	−0.55, −0.015
Age of gaming onset	7.96	2.96	8.80	3.26	2.65	390	0.008	0.27	0.07, 0.47
Weekly gaming hours	12.36	10.47	8.66	8.18	−3.78	392	<0.001	0.38	−0.58, −0.18
Toxicity	2.59	1.39	2.14	1.38	−3.20	392	<0.001	0.32	−0.52, −0.12
Gaming Motivations									
Immersion	14.18	4.12	14.07	4.50	−0.07	392	0.394	0.02	−0.22, 0.17
Customization	12.78	4.42	12.98	4.23	0.44	392	0.660	0.04	−0.15, 0.24
Violent Gratification	12.12	4.35	10.60	4.42	−3.39	392	<0.001	0.34	−0.54, −0.14
Coping	14.06	4.02	12.80	4.41	−2.93	392	0.004	0.29	−0.49, −0.09
Fun	17.58	2.94	17.20	3.15	−1.21	392	0.228	0.12	−0.32, 0.07
Cognitive Challenge	13.44	4.15	12.17	4.14	−3.01	392	0.003	0.31	−0.51, −0.11
Competition	11.72	4.15	11.03	4.03	−1.64	392	0.100	0.17	−0.37, 0.03
Social Interaction	13.34	4.35	12.03	4.69	−2.84	392	0.002	0.29	−0.49, −0.09

*Note.* N men = 228; N women = 166. For age of gaming onset, there was 1 missing value in men and 1 in women. *M:* mean; *SD*: standard deviation; *df:* degree of freedom; *g*: Hedges’ *g*. Motivation subscale comparisons were evaluated against a Bonferroni-corrected threshold (α = 0.05/8 = 0.00625) and a Benjamini–Hochberg FDR procedure; all four significant motivations (violent gratification, coping, cognitive challenge, social interaction) remained significant under both corrections.

**Table 2:** Discriminant analysis results.

Predictors	Structure Coefficients	Standardized Coefficients	Pooled Within-Group Correlations Among Predictors	
			Violent Gratification	Age
Weekly hours of gaming	0.62	0.46	0.134	0.121
Violent Gratification	0.58	0.63		−0.188
Age	0.56	0.63		
Canonical R	0.30			
Eigenvalue	0.10			

**Table 3:** Classification results using leave-one-out cross-validation method.

Sample	Gender	Men (Predicted)	Women (Predicted)	% Correctly Classified
Original	Men	137	91	60.1
	Women	56	110	66.3
	% total cases correctly classified			62.7
Cross-validated	Men	135	93	59.2
	Women	58	108	65.1
	% total cases correctly classified			61.7

## Data Availability

The original contributions presented in this study are included in the article. Further inquiries can be directed to the corresponding author.
